# Diversity of Mosaic *pbp2x* Families in Penicillin-Resistant Streptococcus pneumoniae from Iran and Romania

**DOI:** 10.1128/AAC.01535-17

**Published:** 2017-11-22

**Authors:** Seyed Fazlollah Mousavi, Marina Pana, Mohammad Feizabadi, Pantea Jalali, Maria Ghita, Dalia Denapaite, Regine Hakenbeck

**Affiliations:** a Department of Microbiology, Pasteur Institute of Iran, Tehran, Iran; b Cantacuzino National Institute of Research-Development for Microbiology and Immunology, Bucharest, Romania; c Department of Microbiology, Faculty of Medicine, Tehran University of Medical Science, Tehran, Iran; d Department of Microbiology, University of Kaiserslautern, Kaiserslautern, Germany

**Keywords:** Streptococcus pneumoniae, mosaic genes, penicillin resistance, penicillin-binding proteins

## Abstract

Penicillin-resistant Streptococcus pneumoniae strains are found at high rates in Romania and Iran. The mosaic structure of PBP2x was investigated in 9 strains from Iran and in 15 strains from Romania to understand their evolutionary history. Mutations potentially important for β-lactam resistance were identified by comparison of the PBP2x sequences with the sequence of the related PBP2x of reference penicillin-sensitive S. mitis strains. Two main PBP2x mosaic gene families were recognized. Eight Iranian strains expressed PBP2x variants in group 1, which had a mosaic block highly related to PBP2x of the Spain^23F^-1 clone, which is widespread among international penicillin-resistant S. pneumoniae clones. A second unique PBP2x group was observed in Romanian strains; furthermore, three PBP2x single mosaic variants were found. Sequence blocks of penicillin-sensitive strain S. mitis 658 were common among PBP2x variants from strains from both countries. Each PBP2x group contained specific signature mutations within the transpeptidase domain, documenting the existence of distinct mutational pathways for the development of penicillin resistance.

## INTRODUCTION

The increased rates of occurrence of penicillin- and multiple-antibiotic-resistant Streptococcus pneumoniae strains (referred to here as penicillin-resistant S. pneumoniae [PRSP] strains) worldwide, which have been recognized since the early 1980s, represent a paradigm for the evolution of a pathogen due to the selective pressure of antibiotic use. In 1997, the Pneumococcal Molecular Epidemiology Network (PMEN) was established for the classification of 16 global resistant clones based on multilocus sequence typing (MLST) (1). Currently, 43 such clones have been accepted, and antibiotic-sensitive clones that are important in disease are now also included (http://web1.sph.emory.edu/PMEN/). Spain^23F^-1 is the most successful PRSP clone, and representatives of this clone were first identified in Spain in 1984 (2). Members with the same sequence type (ST) as the Spain^23F^-1 clone, ST81, and related clonal variants that belong to clonal complex 81 have been isolated on every continent since then and reveal substantial genetic variability, including variability in their capsular types, due to the ongoing selective pressures imposed by the use of antibiotics and vaccination strategies (3–5). A likely ancestor of Spain^23F^-1, a penicillin-nonsusceptible pneumococcus from Australia isolated in 1967, was recently discovered, and it was documented that the clone serves as the donor of genes associated with antibiotic resistance and virulence to many other S. pneumoniae clones (6).

Alterations in penicillin-binding proteins (PBPs) are the main cause of penicillin resistance. PBPs, the target enzymes of β-lactam antibiotics, form a rather stable covalent complex via their active-site serine, and these acylated PBPs are enzymatically inactive. Mutations in the PBPs of penicillin-resistant laboratory mutants and clinical strains decrease the affinity to the antibiotic inhibitor molecule, and thus, the strains can still function in the presence of increased drug concentrations. The identification of the amino acid changes involved in resistance has been the focus of multiple investigations, since, in combination with structural information, knowledge of these changes will help to provide an understanding of the enzymatic function of PBPs and might facilitate the design of new inhibitors.

Three of the six pneumococcal PBPs, PBP2x, PBP2b, and PBP1a, act as the main players for the resistance phenotype (for reviews, see references 7 and 8). In PRSP they are encoded by mosaic genes containing highly altered sequence blocks differing by up to 25% from the highly conserved sequences in sensitive strains. They display a highly variable mosaic pattern in genetically distinct resistant PRSP clones (9), aggravating the identification of mutations involved in penicillin resistance. It has long been recognized that the commensal species S. mitis is one of the main donors of such mosaic blocks (10, 11). Four distinct PBP2x variants that account for most of the astounding variation of mosaic PBP sequences among resistant streptococci, including S. pneumoniae and S. pseudopneumoniae, as well as S. infantis and S. oralis, were identified in S. mitis (12, 13). Considering that S. mitis is also a naturally highly penicillin-sensitive species similar to S. pneumoniae, this indicates that mutations relevant for penicillin resistance are first selected in S. mitis prior to the transfer of such ready-made gene sequences into the pathogen S. pneumoniae. Secondary mutations which might be related to an altered resistance spectrum and to PBP function in different genetic environments likely occur. In addition, non-PBP genes, such as *murM* and *ciaH*, also play a role in penicillin resistance, at least in particular PRSP clones. PRSP clones with a mosaic MurM gene produce an altered peptidoglycan containing an increased proportion of indirect cross-links that include branched stem peptides (14). CiaH mutations which occur frequently in laboratory mutants are rare in clinical isolates but contribute to penicillin resistance, especially in combination with altered PBPs (15, 16).

We have focused on alterations in PBP2x for several reasons. PBP2x and PBP2b are primary resistance determinants in which mutations in the protein confer increased resistance levels without changes in other PBP or non-PBP genes (17). Mutations in PBP2x confer resistance to penicillins and cefotaxime, whereas PBP2b does not mediate cefotaxime resistance (18), and mutations in PBP2x are a prerequisite for high-level resistance, which also requires alterations at least in PBP1a, in clinical isolates (19). Moreover, high-resolution crystal structures of PBP2x from penicillin-sensitive and -resistant strains are available (20–22).

This study investigated PBP2x of PRSP clones from Iran and Romania, two countries where PRSP strains are found at extremely high rates. In Iran, 78% of S. pneumoniae isolates were reported to be PRSP in 2001 (23), but the rate declined during the next decade, when rates of between 9% and 60% were reported, depending on the place and site of isolation and the underlying disease (24–26). In Romania, the rate of penicillin resistance among S. pneumoniae isolates was already 93% in the early 1990s (27), and rates of from 70% to over 90% have been reported since then (28–31). One study investigated the PBP2x, PBP1a, and PBP2b sequences of nine isolates with high-level penicillin resistance from Romania, and all isolates were found to contain the same amino acid changes, which were close to active-site motifs (32), but the sequences were not accessible. The mosaic structure and alterations that occur in PBP2x in 9 Iranian and 15 Romanian strains are described here.

## RESULTS

### Strain collection.

The Iranian isolates, obtained from four hospitals in Tehran, Iran, were of serotypes 3, 4, 9V, and 19A and serogroup 6 (Table 1), consistent with the broad range of serotypes and serogroups associated with PRSP strains in that country (24, 25). The sequence type (ST) derived from MLST analysis was obtained for seven isolates and revealed that all had a distinct ST, i.e., that they were not epidemiologically related. Only the two strains IR148 and IR174 are apparently members of the same clone, as revealed by their identical restriction patterns on pulsed-field gel electrophoresis (not shown). IR13 was of ST81 and thus belongs to the clone Spain^23F^-1; IR52 was of ST558, to which serotype 35B isolates from different continents also belong; and all others displayed new STs (Table 1). The serogroup was determined for 11 Romanian isolates: 10 were serogroup 19, which is prevalent in Romania (30, 31), and 1 was serogroup 6.

**TABLE 1:** Properties of the S. pneumoniae strains

Strain	Source^*a*^	Date	Age (yr)	Sex	Site of isolation^*b*^	Serotype or serogroup	ST^*c*^	MIC^*d*^ (μg/ml)				Susceptibility^*e*^					*pbp2x* GenBank accession no.
								PEN	OXA	CTX	AMX	ERY	TET	CAM	GEN	SXT	
IR13	Sina Hospital	2008	68	Male	CSF	6A/B	81					S	S	S	R	S	MF506856
IR52	Roghayyeh Nursery	2011	2	Male	Nasopharynx	9V	558					S	S	R	R	R	MF506857
IR120	Shobeir Nursery	2011	3	Male	Nasopharynx	19A	13331					S	S	S	R	R	MF506858
IR135	Shobeir Nursery	2011	2	Female	Nasopharynx	4	ND^*f*^					R	R	R	R	R	MF506859
IR148	Ameneh Nursery	2011	2	Male	Nasopharynx	3	13387					S	S	S	R	R	MF506861
IR136	Ameneh Nursery	2011	3	Male	Nasopharynx	4	13330					S	S	S	R	R	MF506860
IR158	Roghayyeh Nursery	2011	2	Female	Nasopharynx	6A/B	13329					S	S	S	R	R	MF506862
IR164	Roghayyeh Nursery	2011	0.5	Male	Nasopharynx	6A/B	13328					S	S	S	R	R	MF506863
IR174	Roghayyeh Nursery	2011	1.5	Female	Nasopharynx	ND	ND					S	S	R	R	R	MF506864
RO6	Victor Babes hospital	2004	55	Male	PF	19		4–8	16	0.5	8	R	R	R			MF506865
RO27	Coltea Hospital	2003	79	Female	Sinus	19		4–8	32	0.5	8	R	R	S			MF506866
RO31	Victor Babes Hospital	2004	19	Male	TA	6		4–6	24	0.5	2	R	R	S			MF506867
RO33	Victor Babes Hospital	2003	10	Male	TA			4	24	0.5	8	R	R	S			MF506868
RO34	Victor Babes Hospital	2004	51	Female	PF	19		4–8	16	0.5	8	R	R	S			MF506869
RO36	Marie Curie Hospital	2003	4	Male	TA			1.5–4	12	0.5	2	R	R	S			MF506870
RO56	Coltea Hospital	2004	61	Male	Sputum	19		0.5–4	6	0.5	1	S	R	S			MF506871
RO58	Victor Babes Hospital	2004	12	Female	TA	19		3–4	16	0.5	4	R	R	S			MF506872
RO61	Victor Babes Hospital	2004	46	Male	TA	19		4	24	0.5	4	R	R	S			MF506873
RO67	Coltea Hospital	2004	69	Male	Sputum	19		2–4	8	1	2	S	S	R			MF506874
RO76	Marie Curie Hospital	2004	3	Female	Ear	19		8	48	8	>8	R	S	S			MF506875
RO85	Victor Babes Hospital	2004	7	Female	TA			3–8	24	0.5	2	R	R	S			MF506876
RO94	Marie Curie Hospital	2004	2	Male	TA			4–8	24	4	8	S	S	S			MF506877
RO106	Marie Curie Hospital	2004	5	Male	TA	19		4–8	16	0.5	8	R	R	S			MF506878
RO116	Marie Curie Hospital	2003	1	Female	CS	19		3–4	1.5–2	2	2	S	S	S			MF506879

a The hospitals or nurseries are located in Tehran (Iranian [IR] isolates) or Bucharest (Romanian [RO] isolates).

b TA, tracheal aspirate; PF, pleural fluid; CS, conjunctival secretion; CSF, cerebrospinal fluid; Ear, middle ear fluid.

c ST, sequence type defined by MLST; the pulsed-field gel electrophoresis (PFGE) pattern of strain IR174 was identical to that of strain IR148 (not shown).

d MIC values for β-lactam antibiotics were determined by the agar dilution method; for all other antibiotics, the Kirby-Bauer disk diffusion test was used. All isolates from Iran were resistant to oxacillin and susceptible to cefotaxime, according to published guidelines (61). PEN, penicillin; OXA, oxacillin; CTX, cefotaxime; AMX, amoxicillin.

e ERY, erythromycin; TET, tetracycline; CAM, chloramphenicol; GEN, gentamicin; SXT, trimethoprim-sulfamethoxazole; S, susceptible; R, resistant.

f ND, not determined.

### Mosaic gene families.

**FIG 1:**
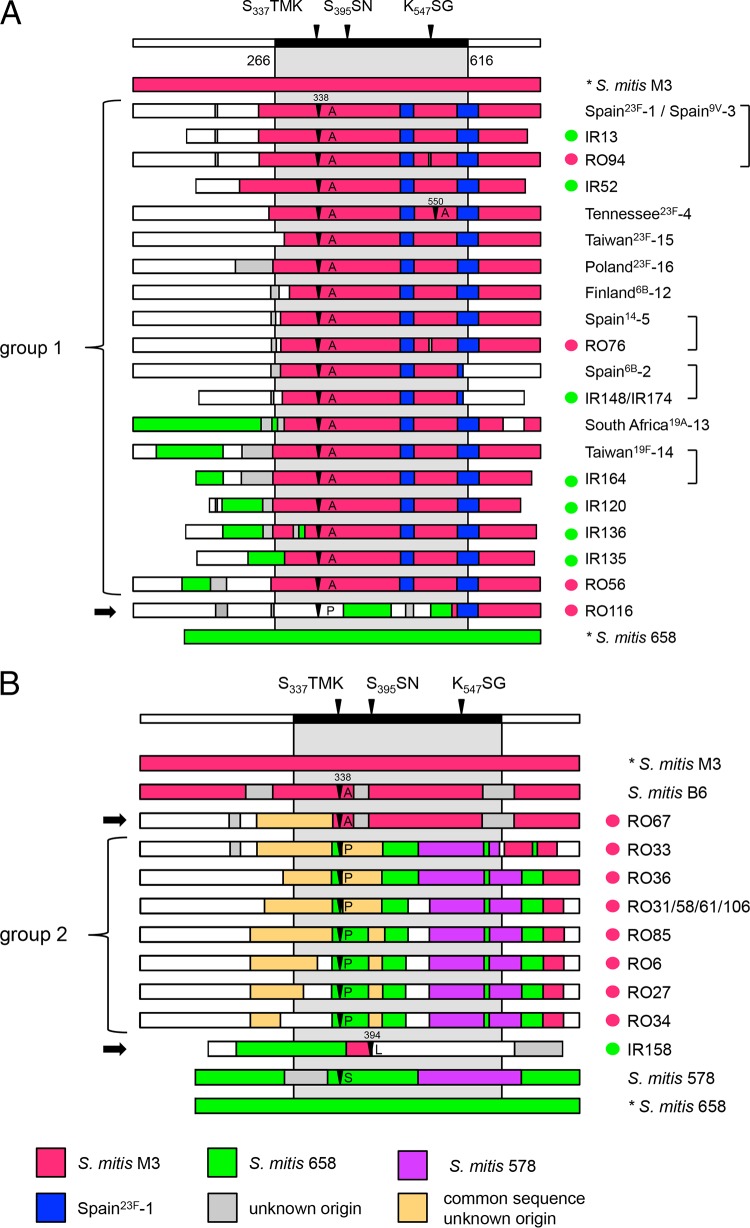
Mosaic structures of the PBP2x variants. Mosaic PBP2x structures were deduced by comparison with the reference PBP2x sequences of S. pneumoniae R6 (white sequence blocks) and S. mitis strains M3 (red sequence blocks) and 658 (green sequence blocks). Highly similar sequences (<5% difference) are shown in the same color; reference sequences are indicated by the color code at the bottom. The active-site motifs of PBP2x within the transpeptidase domain (aa 266 to 616) are shown on top; the gray-shaded areas indicate the central transpeptidase domain. Mutations at sites 338, 394, and 550, close to active-site motifs, are marked by black arrowheads. The strains are indicated on the right. Green dots, strains from Iran; red dots, strains from Romania. Highly similar variants are indicated by brackets on the right; black arrows on the left indicate single PBP2x variants. (A) PBP2x containing sequences similar to those of *pbp2x* of the Spain^23F^-1 clone (group 1) and the single PBP2x variant RO116; (B) group 2 *pbp2x* and the single PBP2x variants RO67 and IR158, including related sequences of S. mitis strains B6 and 578.

The *pbp2x* sequences of the Iranian and Romanian strains were compared to those of the first 16 PMEN clones (1) to see whether they display similar mosaic structures. Two main groups of PBP2x clones were recognized (Fig. 1A and B). Group 1 consisted of the large family of Spain^23F^-1-related *pbp2x* clones, here also referred to as the 23F family (Fig. 1A), which included eight *pbp2x* clones from Iranian strains and three *pbp2x* clones from Romanian strains, and nine PMEN clones. In the Spain^23F^-1 clone, the mosaic block of *pbp2x* covers the central transpeptidase domain (codons 266 to 616 of *pbp2x*) and extends through the 3′ end. This sequence block is highly related to *pbp2x* of the sensitive S. mitis M3 strain first wrongly described as S. oralis, interspersed with two characteristic diverse sequence regions (11) (Fig. 1A). One strain, RO116, included the 23F family block only after codon 586, whereas two S. mitis 658-related sequence blocks were located within the transpeptidase domain after codon 387 (Fig. 1A).

The *pbp2x* genes of six strains of our collection were almost identical to the *pbp2x* genes of four PMEN clones: *pbp2x* of strain IR13, a member of the Spain^23F^-1 clone, was identical to that of the type strain, ATCC 700902, and *pbp2x* of strain RO94 (*pbp2x*_RO94_) was almost identical, differing by 2 nucleotides (nt) and a cluster of 6 nt, resulting in 3 amino acid (aa) changes. These alterations were also present in *pbp2x* of strain RO76 (*pbp2x*_RO76_), which was almost identical to *pbp2x* of the Spain^14^-5 clone (11 nt and 3 aa changes). *pbp2x* of strain IR164 (*pbp2x*_IR164_) was similar to that of the Taiwan^19F^-14 clone (4 nt and 2 aa changes). Finally, the *pbp2x* genes of strains IR148 and IR174 (*pbp2x*_IR148_ and *pbp2x*_IR174_), which were identical to each other, differed from the *pbp2x* gene of the Spain^6B^-2 clone by 18 nt and 4 aa, but only 2 nt and 2 aa changes were located within the common mosaic block.

Noteworthy was the presence of sequences related to the sequence of S. mitis 658 (Fig. 1A, green), one of the penicillin-sensitive reference strains used for the definition of mosaic sequence blocks, mainly in the 5′ region, in seven *pbp2x* variants (those from the South Africa^19A^-13 clone and strains IR164/Taiwan^19F^-14, IR120, IR135, IR136, RO56, and RO116).

The second group consisted of seven variants derived from 10 Romanian strains (Fig. 1B). They all contained sequences related to the *pbp2x* of low-level-resistant S. mitis strain 578 which belongs to the family of mosaic genes related to the *pbp2x* gene of sensitive reference strain S. mitis 658 (12). Moreover, they contained large blocks of identical sequences of unknown origin. This sequence block was also present in the single *pbp2x* variant from strain RO67 (*pbp2x*_RO67_), which was almost identical to *pbp2x* of the high-level-resistant S. mitis B6 strain after codon 329 (4 nt and 3 aa differences). The single *pbp2x* variant from an Iranian strain (strain IR158) contained sequence blocks related only to the sequences of reference S. mitis strains M3 and 658 (Fig. 1B); it was identical to a *pbp2x* gene from Hungarian strain Hu7 of a different ST (not shown).

### Mutations in PBP2x.

**FIG 2:**
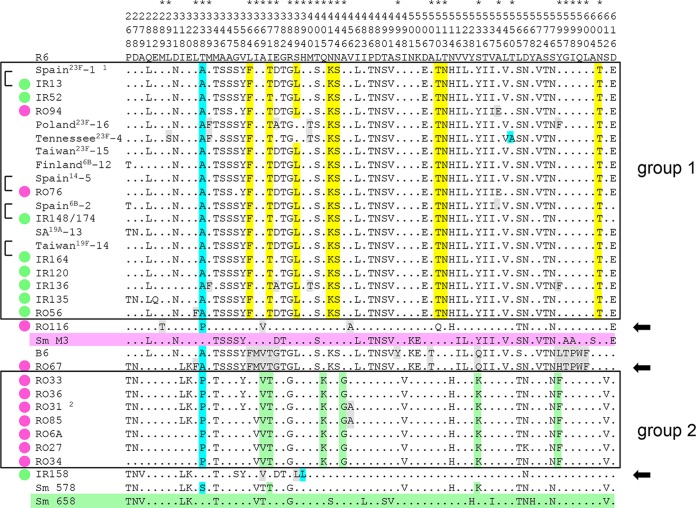
Alignment of PBP2x sequences. Only sites of the transpeptidase domain that differ from PBP2x of S. pneumoniae R6 are shown. The vertical numbers in the first three rows indicate the amino acid positions. The asterisks above the amino acid position mark relevant mutations (see the text for details). The PBP2x variants of group 1 and group 2 are boxed. The color code for the mutations is as follows: light blue, mutations close to active-site motifs (aa 338, 394, and 550); yellow and green, signature mutations of group 1 and group 2 PBP2x, respectively; gray, mutations that occur only in particular variants. The reference sequences of S. mitis (Sm) strains M3 and 658 are shaded in pink and green, respectively. Black arrows on the right mark distinct PBP2x variants; brackets on the left indicate highly similar sequences. Green dots, strains from Iran; red dots, strains from Romania. ^1^, PBP2x of the Spain^23F^-1 clone is identical to PBP2x of the Spain^9V^-3 clone and S. pneumoniae CGSP14 (6); ^2^, a PBP2x variant identical to that in RO31 is present in RO58, RO61, and RO106. SA^19A^-13, South Africa^19A^-13 clone.

The 750-aa PBP2x is a multidomain protein consisting of a short N-terminal membrane anchor, an N-terminal domain, and a central transpeptidase domain (residues 266 to 616), followed by a linker region and a C-terminal extension (residues 635 to 750) which is folded into two PASTA domains (22). Mutations associated with resistance have been described only within the central transpeptidase domain of the protein, which contains three boxes highly conserved in penicillin-sensitive streptococci: S337TMK with the active-site serine, S394SN, and K547TG. Within the transpeptidase domain, a total of 73 sites in all PBP2x variants, including PBP2x of the PMEN clones and S. mitis B6, were distinct from PBP2x of the sensitive S. pneumoniae R6 strain (Fig. 2). However, 41 of them were present in the four penicillin-sensitive reference S. mitis strains M3, 658, SV01, and NCTC10712 (12), leaving 32 sites to be considered. The changes E282Q, N501V, D506E, L510Q, and N514H occurred in PBP2x of S. mitis NCTC10712 or S. mitis SV01, whose sequences are not included in Fig. 2.

All group 1 PBP2x variants except those of the Poland^23F^-16 and Tennessee^23F^-4 clones, where S389 was not altered, contained the signature mutations of this family: T338A, L364F, I371T, S389L, N417K, N444S, L510T, T513N, and N605T (Fig. 2). Other mutations were observed only in some PBP2x variants. L293S and T550A were restricted to the Tennessee^23F^-4 clone, as described previously (33). PBP2x of IR136 contained the additional changes M339F, E378A, M400T, and Y595F, which were also found in PBP2x of the Poland^23F^-16 clone. A545E was present only in the PBP2x variants of Romanian strains RO56 and RO76, and L328F was present in RO56.

PBP2x of strain RO116 (where the mosaic block of the Spain^23F^-1 clone was restricted to the C-terminal end of the transpeptidase domain) contained completely distinct mutations. First, T338 was changed to P, as in PBP2x of all group 2 Romanian strains, and it possessed the three alterations M289T, A369V, and V456A.

Group 2 PBP2x variants shared seven mutations: T338P, A369V, I371T, Q405K, A446G, S531K, and Y595F. Two variants contained V456A (the strain RO31 PBP2x variants and the PBP2x variant of strains RO58, RO61, RO106, and RO85, in which the PBP2x proteins of the last four strains had identical PBP2x sequences).

PBP2x of strain RO67 shared all mutations with PBP2x of S. mitis B6: T338A, I366M, A369V, I371T, E378G, A507T, S531Q, and a cluster of 5 aa changes at sites 595 and 597 to 600. There were three differences between these two PBP2x variants: L328F was present in RO67 and RO56 but not in S. mitis B6, site 595 was changed to L in S. mitis B6 but was Y in RO67, and only PBP2x of S. mitis B6 carried S494Y. PBP2x of IR158 was the only one without a change at site 338, but it contained the mutation H394L, which is close to the second conserved active-site motif S395SN, plus the mutations A369V and S389L.

## DISCUSSION

The present study extends the analysis of mosaic PBP2x structures and mutations from previous analyses performed mainly with commensal streptococci (12) to S. pneumoniae strains isolated from two geographic areas, Romania and Iran. Both countries reported extremely high rates of occurrence of PRSP between 1990 and early in this century, but only limited data on the underlying changes in PBPs are available (32, 34). A detailed analysis of PBP2x, one of the primary targets for β-lactam antibiotics, may act as a start to unravel the evolutionary pathway of penicillin resistance, including the contribution of commensal streptococci to its genetic variation.

### Mosaic structure of PBP2x.

Two large groups of mosaic PBP2x genes are described here. The first group, the 23F family, represented by *pbp2x* of the PRSP clone Spain^23F^-1, included *pbp2x* from Iranian and Romanian strains and 9 of the first 16 PRSP PMEN clones isolated in the 1980s and 1997 (1), confirming its dissemination throughout the pneumococcal population worldwide, as shown previously (6). A total of 17 *pbp2x* variants of group 1 were observed among the 21 strains shown in Fig. 1A. It is of interest that the mosaic block of S. mitis M3-related sequences in *pbp2x* of strain IR52 (*pbp2x*_IR52_) extended into the region encoding the N-terminal domain of the protein farther than in any other variants, a phenomenon observed so far only in PBP2x of penicillin-resistant S. mitis and S. oralis strains (12, 35–37). This suggests that *pbp2x* of IR52 is the result of interspecies gene transfer rather than transfer from another S. pneumoniae clone. Moreover, one strain, strain RO116, contained the Spain^23F^-1-specific mosaic block only after codon 586, but the main parts of the transpeptidase domain were of different origins, with the sequence of this region in RO116 being related to that in S. mitis 658. Short diverse regions whose sequences do not match the Spain^23F^-1 clone or S. mitis 658 sequence frequently flank the 5′ region of the mosaic block, an indication that recombination events contribute to sequence variation (Fig. 1, gray).

The presence of sequence blocks of variable length highly related to the sequence of the sensitive reference strain S. mitis 658, which was common among the *pbp2x* genes of the Iranian strains, documents the presence of donors other than the Spain^23F^-1 clone in this region. Sequences related to those of other *pbp2x* genes from penicillin-sensitive reference strains (S. mitis strains SV01 and NCTC10712, S. infantis JR, and S. oralis ATCC 35037) occur in particular *pbp2x* variants of the 23F family (12). These sequence blocks are frequently located in the regions flanking the transpeptidase domain, suggesting that the signature mutations within the transpeptidase domain are evolutionarily advantageous for PBP function independently of the sequences of the other domains. This and the variability of apparent recombination sites within the 23F family are signs of multiple inter- and intraspecies gene transfer events.

The second group consisted only of PBP2x variants from 10 Romanian isolates that expressed unusually high levels of penicillin resistance and for which penicillin MICs were 4 to 8 μg/ml, a phenotype prevalent in Romania (32). Related *pbp2x* variants were not found in other PRSP strains when the NCBI databases (containing nucleotide and draft genome sequences) were searched by BLAST analysis. Since the isolates were mainly of serogroup 19, it is tempting to assume that they are genetically related.

### Mutations in PBP2x.

**FIG 3:**
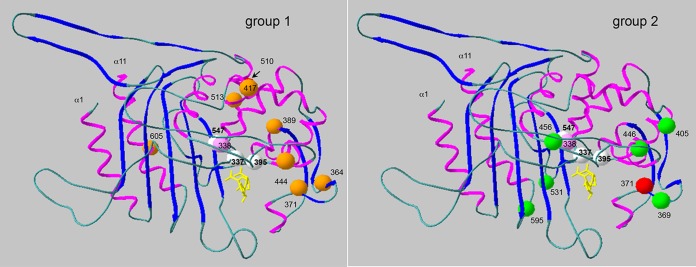
Positions of the signature mutations in group 1 and group 2 PBP2x variants. The structure of the transpeptidase domain of the acylated form of PBP2x of S. pneumoniae R6 with cefuroxime is shown (PDB accession number 1QMF) (20). The positions of the mutations are indicated. Light pink, site 338 close to active-site serine 337; white, active-site residues (S337, S395, and K547); red, position 371, which is common to both protein groups; yellow, the cefuroxime molecule in the active-site cavity.

In each of the two groups of PBP2x variants, distinct sets of mutations which were absent in sensitive S. mitis reference strains were reflected by their different positions within the protein structure (Fig. 3). The signature mutations of the 23F family (F364, T371, L389, K417, S444, T510, N513, and T605) have been described in many publications (9, 11–13, 38–43), consistent with the widespread occurrence of such variants among PRSP strains (6). S389L was absent in two PBP2x variants (from the Poland^23F^-16 and Tennessee^23F^-4 clones), suggesting that it is not important for the resistance phenotype. On the other hand, structural data revealed that the L389 mutation produces a destabilizing effect that generates an open active site (22), similar to the effect of H514, which occurs in the penicillin-sensitive S. mitis strain NCTC10712, and thus might affect the protein function other than the interaction with β-lactams. The only mutation also present in the group 2 PBP2x variants and in S. mitis 578 as well was the change I371T. This mutation results in a slight displacement of the SXN motif, leading to a more accessible active site (22). *In vitro* mutagenesis of the *pbp2x* gene of strain 5204 (*pbp2x*_5204_) in combination with the results of biochemical and microbiological tests confirmed that T371, A338, and T605 are critical for resistance (38).

Some mutations occur only in individual strains. The T550A mutation results in hypersensitivity to penicillins but increased resistance to cefotaxime (44) due to a decreased acylation efficiency for cefotaxime (20, 45), whereas the L393S mutation, which was also present in the Tennessee^23F^-4 clone, has no impact on resistance (33). *In vitro* determination of deacylation and acylation kinetics confirmed that M339F and M400T contribute to resistance (38, 46–48). Y595F occurs in two PBP2x variants of the 23F family (those from the Poland^23F^-16 clone and IR136) and in all group 2 PBP2x variants and is associated with high-level cefotaxime resistance in S. pneumoniae (39). The reversal of F595 to Y595 resulted in a decrease in the level of β-lactam susceptibility (39), but the *in vitro* acylation efficiency was only slightly affected in a mutant containing F595 compared with the acylation efficiency of the wild-type protein (38). This mutation, which lies at the beginning of helix α11 (Fig. 3), has been suggested to be important for β-lactam recognition, similar to other mutations at sites 597 to 600 (20). E378A has been noted in high-level-resistant S. mitis strains (13), and G378 is present in the distinct variants PBP2x_RO67_ and PBP2x of S. mitis B6. The change A545E, present in two Romanian strains, has not been described. The roles of these mutations in resistance will require further analyses.

Of the seven changes highlighted in the group 2 PBP2x variants, only I371T and S531K are present in S. mitis 578, suggesting that the other changes (T338P, A369V, Q405K, A446G, and Y595F) contribute to the high resistance level of the Romanian strains. The mutation A338, which was frequent in the 23F family, reduces the acylation efficiency for cefotaxime (*k*2/*K*, where *k*2 is the rate constant and *K* is the dissociation constant) by a factor of 2, whereas a 5-fold reduction was observed for strains with the P338 mutation (49), consistent with the high penicillin resistance level of strains containing group 2 PBP2x variants. Site 531 is rarely changed to I/K/N/Q/R in resistant commensal streptococci (12, 13), and A446G has been described in a high-level penicillin-resistant S. mitis strain (13). A369V has been identified in S. mitis strains with a benzylpenicillin MIC value of 0.016 μg/ml and thus might not contribute to penicillin resistance (13). Q405K is positioned at the surface of the protein, and thus, its impact on resistance is also questionable. V456A occurred in only two group 2 PBP2x variants and might result in structural changes due to the smaller side chain of the alanine residue.

There were three distinct PBP2x variants outside the two groups described above. PBP2x of strain RO116 contains the mutations T338P, A369V, and V456A present in group 2 PBP2x variants, and M289T has been identified in a cefotaxime-resistant laboratory mutant (44), in agreement with the potential impact of all four mutations on β-lactam resistance. PBP2x of strain RO67 was almost identical to that of the high-level penicillin-resistant strain S. mitis B6, with an accumulation of mutations between sites 595 and 600 which were described in another set of Romanian strains of serotype 23F (32). Strain IR158 contained the only PBP2x variant without a mutation at site 338, but it had A369V, S389L, and H394L. H394L is located next to the conserved motif S395SN and has been described only in low-level-resistant isolates and always without mutations close to the other two conserved motifs at site 338 or 552 (12, 13, 40, 50). The change from histidine, an aromatic, weak basic amino acid, to the aliphatic leucine at a critical position close to the active site of the protein is likely to affect the interaction with β-lactams.

In summary, despite the high variability of mosaic PBP2x genes in PRSP strains, even within groups of closely related variants, mutations potentially relevant for resistance can be highlighted on the basis of a comparison with penicillin-sensitive reference strain S. mitis sequences, as shown here for two families of PBP2x variants. By ignoring changes that occur in these reference S. mitis PBP2x alleles, different mutational pattern become more obvious in groups of related PBP2x alleles. Evidence of multiple mutational pathways in PBP2x for the development of penicillin resistance has been obtained on the basis of a comparison of a large data set consisting of the sequences of PBP2x variants from commensal streptococci, in which five distinct families of mosaic PBP2x genes were described, and all of the families contained PBP2x variants of PRSP strains (12), supporting the conclusion of alternative penicillin resistance mechanisms reached by comparing the crystal structures of group 1 PBP2x variants (22, 38) with the crystal structure of a PBP2x variants with a distinct mosaic makeup (21). Considering the overall mosaic makeup of *pbp2x*, features specific to isolates from one geographic area are apparent, such as the presence of S. mitis 658-related sequence blocks in the 5′ region in *pbp2x* of the Iranian isolates and sequence blocks specific to *pbp2x* of the Romanian isolates. The variability of their mosaic *pbp2x* genes resembles that observed in members of another clone that is also restricted to one geographic area, Hungary^19A^-6 (51, 52). Unusual compositions of mosaic *pbp2x* were also observed in isolates from Japan (12). In most studies in which large numbers of strains have been analyzed, the mosaic structure of PBPs was not investigated in detail (42, 43, 53), and the global presence of the 23F family of PBP2x genes in S. pneumoniae and viridans group streptococci as well has frequently masked the occurrence of other variants and mutations specific to minor *pbp2x* mosaic gene families.

It is clear that the high MICs for β-lactams expressed by many of the strains in the present study cannot be explained solely by PBP2x mutations. A single mutation in PBP2x confers an increase in the level of resistance to cefotaxime, the MIC of which for the sensitive R6 strain is 0.02 μg/ml, of between only 1.5- and 30-fold but is the basis for the higher resistance levels in the context of other mutations in PBP2x and other PBPs as well. Moreover, MurM (54–57) and the histidine protein kinase CiaH (16) contribute to resistance, at least in particular PRSP clones. Other components of the peptidoglycan synthesis machinery are likely to interact with PBPs directly or indirectly and might be modified in response to PBP alterations in penicillin-resistant strains as well. All these facts contribute to the difficulties with the experimental verification of the impact of individual mutations within PBP2x (and other PBPs) on penicillin resistance. The availability of larger amounts of genomic data for PRSP and commensal streptococci will facilitate the gathering of more insight into the complex genetic networking between species and unravel the mutations in components involved in the expression of penicillin resistance.

## MATERIALS AND METHODS

### Characterization of bacterial strains and antibiotic susceptibility testing.

Nine strains isolated in 2011 (8 strains) and 2008 (1 strain) in Tehran hospitals and 15 strains from Romania were included in the present study (Table 1). Serotyping was performed by Neufeld's Quellung reaction using in-house-produced serum and antigens from the Statens Serum Institute, Copenhagen, Denmark, for the Romanian strains and by PCR as described previously (58). Identification by MLST was performed as described previously (59). Allele numbers and sequence types were obtained from the MLST database for S. pneumoniae (https://pubmlst.org/spneumoniae/). Susceptibilities to antibiotics were determined by the agar dilution method recommended by CLSI (60) in the case of β-lactam antibiotics for Romanian isolates, according to the method of Long et al. for Iranian isolates (61), and by the Kirby-Bauer disk diffusion method in all other cases.

### DNA isolation and PCR amplification.

Chromosomal DNAs from streptococci were isolated as described previously (11). PCR products were purified using a JetQuick DNA purification kit (GenoMed). PCRs were performed using either GoldStar *Taq* polymerase (Eurogentec) or DreamTaq polymerase (Fischer) according to the manufacturers' instructions. The PBP2x gene fragments of strains from Iran were amplified with the primers pn2xup and pn2xdown (11) and, in the case of the Romanian strains, the primers PM65 (5′-TACAGATGCAACTTAAACGGTTTTCGCGTG) plus PM80 down (5′-GCAAACCACCAATCATGGCAAGAATCACTAG), and direct sequencing of PCR products was performed with consecutive primers. Other PBP2x genes used in the analysis included those related to mosaic blocks of PBP2x from the Iranian and Romanian isolates present in 10 of the 16 global PRSP clones defined by the PMEN network (1) and in S. mitis, as shown in Fig. 1A and B. These sequences were retrieved from the following (GenBank accession numbers are given in parentheses): from strains S. pneumoniae Tennessee^23F^-4 ATCC 51916 (AM779356) (62), S. mitis 578 (KY292539), and S. mitis 658 (KY292535), from the genomes of clones Spain^23F^-1 (FM211187), Spain^6B^-2 (NC_014498), Spain^14^-5 (FWTC01000077), Finland ^6B^-12 (FWWA01000144), South Africa^19A^-13 (FWSX01000072), Taiwan^19F^-14 (NZ_FWSZ00000000), Taiwan^23F^-15 (FWSS01000062), and Poland^23F^-16 (FWSU01000014), and from S. mitis B6 (NC_013853) and S. mitis M3 (LROV01000013).

### Bioinformatic tools and analysis.

PBP2x sequences were aligned by use of the ClustalX2 program (63) and further processed by the GeneDoc program. The PBP2x gene sequences were aligned with each of the reference sequences. Codon sites were included manually and trimmed by the program Clustal Formatter2 to reveal only sites that differ from the reference sequence, as shown in Fig. 2. Sequence blocks that differed by >5% were defined as distinct sequences, as shown by the different colors in Fig. 1.

### Accession number(s).

 The GenBank accession numbers for the *pbp2x* genes from the strains analyzed in this study are MF506856 to MF506879 (Table 1).
